# Anemia Prevalence and Anthropometric Status of Indigenous Women and Young Children in Rural Botswana: The San People

**DOI:** 10.3390/nu13041105

**Published:** 2021-03-28

**Authors:** Tebogo T. Leepile, Kaelo Mokomo, Maitseo M. M. Bolaane, Andrew D. Jones, Akira Takada, Jennifer L. Black, Eduardo Jovel, Crystal D. Karakochuk

**Affiliations:** 1 Integrated Studies in Land and Food Systems, The University of British Columbia, Vancouver, BC V6T 1Z4, Canada; tebogo.leepile@ubc.ca (T.T.L.); j.black@ubc.ca (J.L.B.); e.jovel@mail.ubc.ca (E.J.); 2 BC Children’s Hospital Research Institute, Vancouver, BC V5Z 4H4, Canada; 3 Letloa Trust, Ghanzi, Botswana; kaelomokomo@gmail.com; 4 San Research Centre, Botswana and the Department of History, University of Botswana, Gaborone, Botswana; Bolaanem@ub.ac.bw; 5 Department of Nutritional Sciences, School of Public Health, University of Michigan, Ann Arbor, MI 48109-2029, USA; jonesand@umich.edu; 6 Centre for African Area Studies, Kyoto University, Kyoto 606-8304, Japan; akiratakad@gmail.com; 7 Department of Food, Nutrition, and Health, University of British Columbia, Vancouver, BC V6T 1Z4, Canada; 8 The Peter Wall Institute for Advanced Studies, University of British Columbia, Vancouver, BC V6T 1Z2, Canada

**Keywords:** anemia, women, children, indigenous, Botswana, San People, hemoglobin, wasting, stunting, underweight

## Abstract

In Botswana, there is limited data available on the health and nutritional status of the San People (also known as the Basarwa or Bushmen), an Indigenous minority group primarily living in the Ghanzi District. Our aim in this study was to assess anemia prevalence among and anthropometric indices of women and young children in Ghanzi District through a cross-sectional survey. We recruited 367 mother–child pairs (women 15–49 years and children 6–59 months) in nine randomly selected areas. A capillary blood sample was collected, and weight and height were measured. Hemoglobin (Hb) concentration was measured with use of a hemoglobinometer (HemoCue, AB), as per global recommendations. Overall, adjusted anemia prevalence was 12% in non-pregnant women (Hb < 120 g/L), 26% in pregnant women (Hb < 110 g/L), and 42% in children (Hb < 110 g/L), but it varied widely depending on whether or not the controversial factor of ethnicity was adjusted for (range of 6–26%, 22–30%, and 35–68% prevalence, respectively). Thirty-nine percent (*n* = 133/344) of non-pregnant women and 52% (*n* = 12/23) of pregnant women were underweight (BMI < 18.5 kg/m^2^). In children aged 6–23 months, 41% were underweight (weight-for-age *z*-score < −2 SD), 13% were wasted (weight-for-height *z*-score < −2 SD), and 65% were stunted (height-for-age *z*-score < −2 SD); in children aged 24–59 months, 57% were underweight, 13% were wasted, and 66% were stunted. Fifty-six percent (*n* = 205/367) of women self-reported smoking in any form (rolled cigarettes or snuffing). The high prevalence of smoking among women, underweight status among pregnant women, and anemia, stunting, and wasting among children is of the highest concern for public health and should be addressed in future health and nutrition programming.

## 1. Introduction

Anemia reduction among women of childbearing age and young children in low- and middle-income countries is a significant global health priority [1,2,3,4,5]. In women of reproductive age, anemia is associated with adverse birth and maternal outcomes, reduced productivity, and impaired cognitive and motor development in childhood [6,7,8]. Although relatively few studies have assessed the prevalence of anemia in Indigenous communities, a disproportionate burden of anemia is thought to exist among Indigenous peoples compared to non-Indigenous populations worldwide [9,10,11]. In Botswana, little is known about anemia prevalence or the nutritional status of the San People, an Indigenous minority group [12,13,14] that resides mostly in the Kalahari Desert in Ghanzi District [15,16,17]. The use of the term Indigenous in Botswana is debatable since it is not exclusively ascribed to the San People or any tribal group; instead, the government argues that all Batswana are Indigenous. In this paper the San People are categorized as Indigenous based on the guidance from key national and global frameworks; locally, the Remote Area Development Programme (RADP) recognizes the San People’s ethnic distinctiveness and vulnerability due to their rural habitation, which aligns with some of the crucial tenets of the modern and inclusive understanding of the term “Indigenous” [12,13,14]. Globally, Botswana is a signatory to the United Nations Declaration on the Rights of Indigenous Peoples (UNDRIP), which mandates governments to respect and protect the rights of all Indigenous peoples and regularly participates in the annual United Nations Permanent Forum on Indigenous Issues. Some San groups, for example New Xade and Bere residents, argue that they were illegally removed from their ancestral lands in the Central Kalahari Game Reserve (CKGR) and other places. To date, some international human rights groups have contended that how the San were removed violated international human rights laws [15,16,17]. The San comprise groups of Indigenous peoples of Southern Africa whose main means of subsistence, until recently, has been hunting and gathering. The origins of the term ”San” can be traced back to the designation bestowed by the KhoeKhoe (also spelled as KhoiKhoi), a neighboring ethnic group whose livelihood consists mainly of herding livestock [18]. The San are also referred to as “Bushmen” (a designation given by Europeans) or Basarwa (a designation given by Tswana people, which etymologically means “people of the south”) [19]. Although referred to as San, Bushmen, and Basarwa, this paper employs ”San” as the preferred term of designation, this being the most commonly used term in the context of contemporary international politics and research [18].

Anemia has a myriad of causes including micronutrient deficiencies (e.g., folate, iron, riboflavin, vitamins A and B12), acute and chronic infections (e.g., malaria, tuberculosis and HIV), and inherited genetic disorders (e.g., hemoglobinopathies) [2,3]. Suboptimal nutrition and poor nutritional status heighten the risk of mortality and other related co-morbidities. Childhood undernutrition and micronutrient deficiencies also contribute to delayed growth, impaired cognitive and motor development, and compromised academic ability, which results in increased disease susceptibility and lowers economic productivity and status in adulthood [20,21]. Suboptimal maternal nutrition has the potential to alter fetal programming (a critical process of organ and tissue development), resulting in metabolic syndromes in adulthood [22,23], and is also associated with low-birth weight, preterm birth, and perinatal and neonatal mortality [24].

Some studies conducted in Botswana have shown that anemia and poor nutrition status may be due to HIV status and inadequate diets [25,26,27]; however, no published studies exist that specifically focus on the Indigenous minority group the San People. The lack of information on the health and nutritional status of the San People hampers the development of appropriate and evidence-based health and nutrition interventions and policies. Moreover, the lack of consistent and timely national health surveillance and the general lack in the use of disaggregated data (which entails a failure to carefully examine the data to understand the distribution of risk factors among specific sub-groups, such as the San) exacerbate the problem of not identifying the specific health issues of sub-groups, such as the San.

The aim of this cross-sectional study was, therefore, to assess the prevalence of anemia and the anthropometric indices of San women of childbearing age and young children.

## 2. Materials and Methods

### 2.1. Study Location

This study was conducted in the Ghanzi/Gantsi District (GD) in western Botswana, a region that covers an area of ~117,910 km^2^ at an elevation of ~1156 m (3793 ft) above sea level. GD predominantly consists of rural villages and settlements spread across the Central Kalahari Game Reserve and a peri-urban administrative center, the Ghanzi township. The district’s economy is largely reliant on farming, tourism, and service provision; the region is the nation’s largest beef supplier. Different ethnic groups reside in the GD, including the San (G|ui, G||ana, Naro,!*Xoõ)*, Afrikaners, Bakgalagadi, and Baherero. A significant proportion of the San People reside in the rural areas while some live in the Ghanzi township and inside the Central Kalahari Game Reserve.

### 2.2. Study Design

This study was a cross-sectional survey of San mother–child pairs conducted between March and September 2019. Ethics approval was obtained from the Clinical Research Ethics Board at the University of British Columbia (H17-01696), the Botswana Ministry of Health and Wellness (HPDME 13/18/1), and the University of Botswana (UBR/RES/IRB/BIO/071).

### 2.3. Sampling Method, Eligibility Criteria, and Recruitment

A sample of 367 households and the same number of mother/guardian–child pairs were recruited to participate in this study. Recruitment and community sensitization were primarily carried out in collaboration with the Ghanzi District Health Management Team, traditional community leaders, and influential community members through word of mouth. Awareness was also raised through printed advertisements at health posts and meetings at public gathering places (such as health posts or *kgotlas*; a *kgotla* is a public and community assembly place in Botswana which usually allows the leaders (chief or headman) and the community members to discuss and collectively make decisions about important matters).

One township and eight settlements across geographical areas in GD where the San People reside were randomly selected: Ghanzi, D’kar, Qabo, Grootlaagte, East Hanahai, West Hahahai, Bere, Kaqgae, and New Xade. A list of eligible children (<5 years) was obtained from the Child Welfare Clinics (CWC) through the growth monitoring program (GMP) at each location. This list was subsequently used to select households based on a convenience sampling technique.

The inclusion criteria for both mothers and children were that they were of San origin/heritage and eligible age (mother/guardian 15–49 years, child 6–59 months), whilst mothers/guardians had to have had or cared for at least one child aged 6–59 months, lived in the selected settlement for at least 1 year, and consented to participate in the study. Where there was more than one eligible child (aged 6–59 months) in a household, the youngest child was selected for participation. In addition, the mothers/guardians were also asked to also complete a questionnaire on demographic, health, and other relevant household and socio-economic data.

#### 2.3.1. Anthropometry

Anthropometric measurements were conducted by trained research assistants using calibrated equipment (Seca 213 Stadiometre and Seca 417 Measuring Board for Infants, Health-O-Meter Professional 160 LB Mechanical Floor Medical Scale, Escali All Metal Hanging Scale for Children, and Health-O-Meter 549 KL Digital Baby Scale). Weight and height were measured twice (to the nearest 1.0 cm and 100 g, respectively) and the mean was recorded as the final value. Participants were asked to wear light clothing and no shoes for the measurements. Children’s anthropometric measurements (weight and height/length) were converted to z-scores [28] with the use of the Stata igrowup package [29]. BMI was calculated for women based on measured weight and height measurements (kg/m^2^) and categorized following the World Health Organization (WHO) guidelines: underweight <18.5, normal weight 18.5–24.9, overweight 25.0–29.9, and obese >30.0 kg/m^2^. Childhood malnutrition was defined in accordance with the WHO growth reference as underweight for a weight-for-age z-score (WAZ) < −2 SD; wasting for a weight-for-length (WLZ) or weight-for-height (WHZ) z-score < −2 SD; and stunting for a length-for-age (LAZ) or height-for-age z-score (HAZ) < −2 SD. Extreme outliers (implausible z-score values) were excluded based on UNICEF/WHO guidelines for HAZ (−6, +6 SD), WHZ (−5, +5 SD), and WAZ (−6, +5 SD) [28].

#### 2.3.2. Blood Collection, Processing and Analysis

Capillary blood samples were collected and analyzed for haemoglobin (Hb) concentration in the field with a HemoCue instrument (HemoCue 301, Angelholm, Sweden). The HemoCue is a portable, easy-to-use, factory-calibrated device that is convenient for fieldwork as it only requires a capillary finger-prick blood sample. Blood collection techniques were deployed according to globally endorsed standards [30]. All blood samples were safely discarded immediately after taking the reading in accordance with the global standards and the Canada Tri-Council Policy Statement [31].

Anemia prevalence was calculated with use of Hb concentration cut-offs defined by the WHO thresholds for children (Hb < 110 g/L), pregnant women (Hb < 110 g/L), and non-pregnant women (Hb < 120 g/L) [32]. Hb concentrations were adjusted for altitude, ethnicity, and smoking and for all these factors combined, as per global recommendations [33]. GD is located at an altitude of ~1156 m above sea level, therefore Hb values were adjusted downwards by 2 g/L for all study participants. The San People are of African origin, therefore, the Hb values were adjusted upwards by 10 g/L for all study participants following Sullivan et al. [33]. Lastly, Hb values were adjusted downwards by 3 g/L for women who self-reported smoking in any form and for any duration. Hb concentration and anemia prevalence were reported as unadjusted, altitude-adjusted, ethnicity-adjusted, smoking-adjusted, and adjusted for all factors [33]. Anemia was also categorized based on severity, using WHO recommended categories, for non-pregnant women (mild 110–119 g/L, moderate 80–109 g/L, and severe <80 g/L), for pregnant women (mild 100–109 g/L, moderate 70–99 g/L, and severe <70 g/L), and for children aged 6–59 months (100–109 g/L, moderate 70–99 g/L, and severe <70 g/L) [32].

Data was stratified by rural vs. peri-urban regions, age category, and pregnancy status because of the known existing health disparities between geographical areas and sub-group populations [34,35]. It was also important to stratify data by pregnancy status and age for children in line with the global impetus for optimal health and nutrition for the first 1000 days, a critical window of opportunity for optimal early childhood development [36,37]. Additionally, some studies have identified age as an important predictor of anemia [38,39].

Continuous, normally-distributed variables, for example, age and Hb concentrations, were compared across categories with the use of *t*-tests, and comparisons between binary variables such as sex, anemia status, and smoking were conducted using Pearson chi-square tests. All analyses were conducted in Stata (StataCorp, College Station, TX, USA).

## 3. Results

### 3.1. Household Characteristics

Table 1 provides a summary of household and mother/caregivers’ characteristics. Among women who had previously given birth, the mean ± SD number of children born (parity) was 3.3 ± 2.1 children. Smoking was prevalent among women; 57% in rural areas vs. 51% in peri-urban areas. Households in rural areas were larger than those in the peri-urban areas (8.6 ± 4.5 vs. 7.7 ± 3.9 members). The majority of households had an income < $175 USD per month (69%), while less than 30% households had an income >$176 USD per month. Women in rural areas most commonly reported a secondary school education or higher education status (38%), whereas their counterparts in the peri-urban areas more commonly had primary school education (39%). A large proportion of women were employed (79%); however, the majority were employed in a Public Works Programme (drought relief program) commonly known as Ipelegeng, which offers short-term employment at monthly intervals for a monthly wage of about $45 USD. Water connection in homes was 34%, while 34% and 33% of households sourced water from the public pipes and their neighbors, respectively. Open defecation is a common problem in the region (69%); 83% and 65% in peri-urban and rural areas, respectively.

### 3.2. Anemia Prevalence, BMI, and the HIV Status of Sampled San Women in Rural Botswana

The age, Hb concentrations, BMI, HIV status, and anemia prevalence among enrolled San women are presented in Table 2. The mean ± SD age of women was 29.3 ± 7.3 years. Approximately 45% (*n* = 148/344) of non-pregnant women had a normal BMI (18.5–24.9 kg/m^2^) while 39% (*n* = 133/344) were underweight (BMI <18.5 kg/m^2^). Although BMI is not an accurate index of nutritional status during pregnancy (due to the bias of expected pregnancy weight gain), we found that an alarming 52% of pregnant women were underweight. In comparison with the 2019 estimated national HIV prevalence of 20.3% (25.1% in women aged 15–49 years), 14% of all the San women were infected; 15% among non-pregnant and 4% among pregnant women [40]. Approximately 9% of pregnant women did not know their HIV status compared to 6% of non-pregnant women.

Overall anemia prevalence among all women was 13% after adjusting for altitude, ethnicity, and smoking. A higher proportion of pregnant women were anemic (26%) compared with non-pregnant women (12%). Prevalence varied widely depending on whether Hb was adjusted for altitude, ethnicity, and smoking, or adjusted for all factors combined (Table 2). Overall, rates of anemia among women were higher in peri-urban areas compared to rural areas (20% vs. 11%).

### 3.3. Prevalence of Childhood Malnutrition and Anemia among Children (6–23 and 24–59 Months)

The children’s anthropometric measures (underweight, wasting, and stunting) and anemia prevalence data by region (rural vs. peri-urban) are presented in Table 3. Underweight prevalence among children aged 24–59 months was significantly higher compared to children aged 6–23 months (57% vs. 41% respectively, *p* < 0.0019). Although there was a similar burden of wasting for the two groups (13%), younger children (6–23 months) in the Ghanzi township were affected the most (16% vs. 12% in rural areas). Stunting prevalence was high in all age groups: 65% among children aged 6–23 months and 66% among children aged 24–59 months. However, a higher proportion of younger children (76%) in the peri-urban area were stunted compared to ~60% in rural areas and older children in both rural (66%) and peri-urban areas (65%).

The average unadjusted Hb concentration for all children was 103 ± 15 g/L. Children in peri-urban areas had lower concentrations than their counterparts in rural areas (100 ± 13 vs. 104 ± 16 g/L). Prevalence varied widely depending on whether Hb was adjusted for altitude and ethnicity or adjusted for both factors (Table 3). Anemia prevalence ranged between 42% and 63% after adjustments, as indicated in Table 3.

### 3.4. Severity of Anemia

We also assessed whether the anemia was classified as mild, moderate, or severe, based on WHO categorization [32]. Of the non-pregnant women with anemia (*n* = 84, based on Hb adjusted for all factors) the majority (10%) had mild anemia, 2% had moderate anemia, and <1% had severe anemia. Of the pregnant women with anemia (*n* = 7, based on Hb adjusted for all factors), proportions of mild and moderate anemia were equal at 13%, and there were no cases of severe anemia. Of the children with anemia (*n* = 153, based on Hb adjusted for both altitude and ethnicity), 22% had mild anemia, 19% had moderate anemia, and 1% had severe anemia.

## 4. Discussion

To our knowledge this is the first study to provide comprehensive health and nutrition data on the Indigenous San People of Ghanzi District. Overall, we found that anemia ranged widely depending on Hb adjustment factors, with 6–26% in non-pregnant women, 22–30% in pregnant women, and 35–63% in children. There is a global consensus concerning the adjustment of Hb values for altitude and smoking; however, whether or not an adjustment for ethnicity is warranted remains controversial. Currently, the WHO is in the process of reviewing and updating the guidelines for the use and applicability of Hb cut-offs to define anemia in different populations [41]. Following adjustments for ethnicity, anemia prevalence declined significantly (women: from 22% to 7%; children: from 63% to 35%). Regardless of adjustment factors, anemia prevalence among children was high and of concern.

Given the high prevalence of anemia among both women and children, we also assessed whether or not the proportion of anemia in each group was mild, moderate, or severe. In terms of the public health policy implications, this is an important consideration. Overall <1% of the anemia was categorized as severe. Although reassuring, this is still an important consideration to take into account for the meant and/or prevention of mild/moderate anemia, and thus in improving health status and preventing the possible decline to severe status.

We also observed noteworthy differences for women and children living in the rural and peri-urban areas of our study. Overall, rates of anemia were higher in peri-urban areas, as compared to rural areas, for both women (20% vs. 11%) and children (51% vs. 40%). Typically, malnutrition has been found to be higher in rural areas compared to peri-urban or urban settings. Assessing and reporting specific information per region is critical for guiding policy and enacting appropriate interventions that identify, target, and prioritize the most vulnerable populations and key areas of focus. According to the WHO, in 2011, 43% of Batswana children aged 6–59 months had anemia classified as “severe”, this being judged to be a public health concern; 28% of non-pregnant women were anemic (1.5% had severe anemia); and the prevalence among pregnant women was 32% (0.5% severe anemia) [2]. The situation of pregnant and non-pregnant women was categorized as of moderate public significance. Further comparing our data with the World Bank national data for Botswana for 2016, our study indicated a relatively similar anemia prevalence among San children <5 years (40% vs. 42%, respectively) but higher anemia prevalence among women (30% vs. 13%, respectively). In the same report for 2016, the World Bank estimated a higher national prevalence among both pregnant and non-pregnant women compared to our study; 30% vs. 12% in non-pregnant women and 34% vs. 26% among pregnant women [42]. Several factors, including blood collection techniques (venous and arterial), time of blood collection, and body position, can affect Hb measurement, which might explain some of the observed variations [43]. These results also correlate with existing findings that indicate a persisting problem of anemia across Africa (highest in West and Central Africa), especially among women of reproductive age and children. In 2011, anemia prevalence in Southern Africa (where Botswana is located) was estimated at 46% among children <5 years and 28% and 31% in non-pregnant and pregnant women, respectively [1]. Although the WHO attributes over 50% of anemia cases to iron deficiency due to poor dietary intakes [2], the literature indicates that infections, genetic disorders, and other micronutrient deficiencies in the region also play a significant role [44,45,46,47].

The results also indicate a concerning prevalence of undernutrition among San women and young children. About 39% of non-pregnant women were underweight. Growth faltering indicators among children were also observed, namely stunting (65%, 6–23 months and 66%, 24–59 months), wasting (13% in both age groups), and being underweight (41%, 6–23 months and 57%, 24–59 months). Differences existed between the age groups; children aged 6–23 months in peri-urban settings were the most affected compared to their counterparts in rural areas (stunting, 61% VS. 76%). Conversely, in the older group (24–59 months), the prevalence of child malnutrition was higher among children in rural areas. Based on the WHO cut-off values for public health significance for the child growth indices, the situation in GD warrants attention, as indicated by the findings for being underweight (very high prevalence; ≥30%), wasting (serious; 10–13%), and stunting (very high prevalence; ≥40%) among the two age groups (6–23 months and 24–59 months) [48]. The United Nations Children’s Fund (UNICEF) Botswana in collaboration with the Ministry of Health and Wellness (MoH) assessed the determinants of undernutrition amongst young children (<5 years) in five districts of Botswana in 2015. The Ghanzi region (38.8%) had the highest prevalence of childhood undernutrition compared to the other areas, Francistown (21.6%), Kgalagadi South (19.5%), Kweneng East (29.9%), and Selibe-Phikwe (26.2%) [49].

Another study in the same year that assessed the child feeding practices among young children (2 y) following the ProPan (Process for the Promotion of Child Feeding) approach also confirmed a critical burden of undernutrition among children in the Ghanzi district. Prevalence of wasting (acute malnutrition, weight-for-age *z*-score < −2 SD) was 9% on average and 21% in rural areas. These were above the WHO recommended prevalence cut-off values for public health significance (<5%). Children in rural areas (farms and settlements) were at an increased risk compared to those in the township. A widespread prevalence of sub-optimal infant and young child feeding practices was reported, as evidenced by low rates of breastfeeding, lack of diverse diets, and high food prices. Insufficient incomes due to unstable employment in low-wage jobs might explain the high prevalence of undernutrition in the region [50]. Despite the limited data on the San People, between 1988 and 1999 Kent and Dunn suggested a notable increase of anemia amongst the group due to changes in dietary patterns [27]. Shortly after the relocation from the CKGR, Takada et al. in 2002 reported declines in weight gain in young San children (<1 y) alongside lifestyle transitions characterized by decreased foraging and increased uptake of agricultural activities [51]. There is a paucity of health data on Africa’s Indigenous Peoples; however, data from other countries indicate that Indigenous communities are grappling with undernutrition and micronutrient deficiencies [52,53,54,55,56]. According to the 2007 Botswana Family Health Survey (BFHS), 25.9% of children under 5 years were stunted, 13.5% were underweight, and 7.2% were wasted [57,58].

We can highlight some strengths and limitations of our research. This study provides important and comprehensive information on anemia among and the anthropometric health of the San People and addresses the existing gap in food and nutrition health data in the country. A large number of representative households (*n* = 367 total) were included in our cross-sectional survey. In addition to assessing the anemia prevalence and nutrition status, we also collected other relevant data to help us better understand our findings and assess the health and nutritional context of the San People. One strength was our comprehensive and thorough assessment of anemia status, considering the adjustment factors that are warranted on this population. We acknowledge that we had limited data on the duration and form of smoking, as this exposure is difficult to accurately measure in populations who use various forms and methods (e.g., snuffing). Thus, we conservatively adjusted for smoking according to global recommendations; however, we acknowledge that the effect of smoking on Hb values may be underestimated in our analyses due to this. Dietary data and other information on the other possible causes of anemia would have been helpful. It is important to examine and understand the causes of anemia among the San People in future research and determine whether anemia is caused by low iron intake or other micronutrient deficiencies, diseases, and/or genetic disorders in order to design appropriate interventions to reduce, treat, and prevent anemia.

## 5. Conclusions

The high prevalence of smoking among women, underweight status among pregnant women, and anemia, stunting, and wasting among children is of high concern from a public health perspective. Holistic approaches to improving livelihoods and household incomes are a crucial investment. Adjustments for different factors (altitude, ethnicity, and smoking) yielded different results, with varied public health significance—thus, further research on the adjustment of individuals of ethnic origin is warranted. Future research should focus on determining the causes of anemia and underweight status in this population, as well as exploring strategies to mitigate anemia, malnutrition, and smoking prevalence in this population.

## Figures and Tables

**Table 1:** Descriptive characteristics of enrolled San women in rural Botswana.

	All	Rural	Peri-Urban
Total, *n* (%)	367 (100%)	296 (81%)	71 (19%)
Age, mean ± SD	29.3 ± 7.3	29.3 ± 7.4	28.0 ± 5.2
Parity, mean ± SD	3.3 ± 2.1	3.3 ± 2.1	3.4 ± 2.3
Smoking ^1^, *n* (%)	205 (56%)	169 (57%)	36 (51%)
Household size, mean ± SD	8.5 ± 4.4	8.6 ± 4.5	7.7 ± 3.9
Household monthly income, *n* (%)			
<$175 USD	253 (69%)	208 (70%)	45 (63%)
$176–350 USD	104 (28%)	78 (26%)	26 (36%)
>$350 USD	10 (3%)	10 (3%)	0 (0%)
Mother’s education level, *n* (%)			
No schooling	99 (27%)	75 (25%)	24 (34%)
Pre-school	3 (1%)	2 (1%)	1 (1%)
Primary school	120 (33%)	92 (31%)	28 (39%)
Secondary school	131 (36%)	113 (38%)	18 (25%)
Tertiary/higher education	12 (3%)	12 (4%)	0 (0%)
Other	2 (1%)	2 (1%)	0 (0%)
Employment status, *n* (%)			
Unemployed	76 (21%)	72 (24%)	4 (6%)
Employed	291 (79%)	224 (76%)	67 (94%)
Occupation, *n* (%)			
Drought relief program	282 (77%)	218 (74 %)	64 (90%)
None	76 (21%)	72 (24%)	4 (6%)
Paid Internship	3 (1%)	3 (1%)	0 (0%)
Other	6 (2%)	3 (1%)	3 (4%)
Drinking water source, *n* (%)			
Home-connected pipes/tap	123 (34%)	96 (33%)	27 (38%)
Public pipes/tap	123 (34%)	123 (42%)	0 (0%)
Neighbors’ pipes/tap	121 (33%)	77 (26%)	44 (62%)
Toilet facility, *n* (%)			
No-facility (open defecation)	252 (69%)	193 (65%)	59 (83%)
Flush toilet	14 (4%)	5 (2%)	9 (13%)
Traditional pit latrine	26 (7%)	26 (9%)	0 (0%)
Ventilated improved pit latrine (VIP)	50 (14%)	47 (16%)	3 (4%)
Composing toilet	7 (2%)	7 (2%)	0 (0%)
No facility + VIP latrine	18 (5%)	18 (6%)	0 (0%)
IFA supplementation (any dosage)	33 (9%)	27 (9%)	6 (8%)

^1^ Proportions reflect women who self-reported to smoking for any duration or form (rolled cigarettes, snuffing (orally or nasally)). IFA, iron and folic acid.

**Table 2:** Anemia prevalence, BMI, and HIV status of enrolled San women in rural Botswana ^1^.

	All	Non-Pregnant	Pregnant
Total enrolled	367 (100%)	344 (94%)	23 (6%)
Age, years	29.3 ± 7.3	29.3 ± 7.4	28.0 ± 5.2
BMI, kg/m^2^			
Normal (>18.5 and <24.9)	153 (42 %)	148 (43%)	5 (22%)
Underweight (<18.5)	145 (40%)	133 (39%)	12 (52%)
Overweight (>25 and <29.9)	40 (11%)	35 (10%)	5 (22%)
Obese (>30)	29 (8%)	28 (8%)	1 (4%)
HIV status			
HIV positive	52 (14%)	51 (15%)	1 (4%)
HIV negative	286 (78%)	266 (77%)	20 (87%)
Unknown status	23 (6%)	21 (6%)	2 (9%)
Undisclosed status	6 (2%)	6 (2%)	0 (0%)
Hemoglobin concentration, g/L			
Hb unadjusted	131 ± 16	132 ± 15 ^a^	116 ± 15 ^b^
Hb adjusted for altitude	129 ± 16	130 ± 15 ^a^	114 ± 15 ^b^
Hb adjusted for ethnicity	141 ± 16	142 ± 15 ^a^	126 ± 15 ^b^
Hb adjusted for smoking	129 ± 16	130 ± 15 ^a^	114 ± 15 ^b^
Hb adjusted for altitude, smoking, and ethnicity	137 ± 16	138 ± 15 ^a^	122 ± 15 ^b^
Anemia prevalence, Hb < 110 g/L			
Hb unadjusted	81/367 (22%)	74/344 (22%) ^a^	7/23 (30%) ^a^
Hb adjusted for altitude	96/367 (26%)	89/344 (26%) ^a^	7/23 (30%) ^a^
Hb adjusted for ethnicity	27/367 (7%)	22/344 (6%) ^a^	5/23 (22%) ^b^
Hb adjusted for smoking	91/367 (25%)	84/344 (24%) ^a^	7/23 (30%) ^a^
Hb adjusted for altitude, smoking, and ethnicity	48/367 (13%)	42/344 (12%) ^a^	6/23 (26%) ^a^

^1^ Values are mean ± SD or *n* (%). Hb, hemoglobin. Mean Hb concentrations were compared between pregnant and non-pregnant women using *t*-tests. Comparisons between binary variables were analyzed using Pearson chi-square tests. Hb concentrations were adjusted based on global recommendations [33]. Different superscript letters in the same row indicate statistical differences between pregnant and non-pregnant women (*p* < 0.05).

**Table 3:** Prevalence of childhood malnutrition and anemia among San children (6–23 and 24–59 months).

	All	Rural	Peri-Urban
Total, *n* (%)	367 (100%)	296 (81%)	71 (19%)
Age, months, *n* (%)			
6–23 months	182/367 (50%)	139/296 (47%)	43/71 (61%)
24–59 months	185/367 (50%)	157/296 (53%)	28/71 (39%)
Anthropometrics (Excluded Outliers)			
Underweight, WAZ < −2SD (2 missing) ^1^			
6–23 months	74/182 (41%)	50/139 (36%) ^a^	24/43 (56%) ^b^
24–59 months	104 /183 (57%)	90/156 (58%) ^a^	14/27 (52%) ^a^
Wasted, WHZ < −2SD (4 missing) ^2^			
6–23 months	24/181 (13%)	17/138 (12%) ^a^	7/43 (16%) ^a^
24–59 months	24/181(13%)	21/154 (14%) ^a^	3/27 (11%) ^a^
Stunted, HAZ < −2SD (15 missing) ^3^			
6–23 months	114/176 (65%)	83/135 (61%) ^a^	31/41 (76%) ^a^
24–59 months	115/175 (66%)	98/149 (66%) ^a^	17/26 (65%) ^a^
Hemoglobin concentration, mean ± SD, g/L			
Hb unadjusted	103 ± 15	104 ± 16 ^a^	100 ± 13 ^b^
Hb adjusted for altitude	101 ± 16	102 ± 16 ^a^	98 ± 13 ^b^
Hb adjusted for ethnicity	113 ± 16	114 ± 16 ^a^	110 ± 13 ^b^
Hb adjusted for all (altitude, ethnicity)	111 ± 16	112 ± 16 ^a^	108 ± 13 ^b^
Anemia prevalence Hb < 110 g/L (*n*, %)			
Hb unadjusted	232/367 (63%)	177/296 (60%) ^a^	55/71 (77%) ^b^
Hb adjusted for altitude	250/367 (68%)	192/296 (65%) ^a^	58/71 (82%) ^b^
Hb adjusted for ethnicity	128/367 (35%)	99/296 (33%) ^a^	29/71 (41%) ^a^
Hb adjusted for all (altitude, ethnicity)	153/367 (42%)	117/296 (40%) ^a^	36/71 (51%) ^a^

A total of 21 extreme outliers were excluded and the missing data were as follows: underweight; weight-for-age *z*-score (WAZ) < −2 SD, ^1^ (*n* = 2 excluded), wasting; weight-for-length (WLZ) or weight-for-height (WHZ) *z*-score < −2 SD, ^2^ (*n* = 4 excluded, and *n* = 1 missing length value), stunting; length-for-age (LAZ) or height-for-age *z*-score (HAZ) < −2 SD, ^3^ (*n* = 16 missing, *n* = 15 excluded, *n* = 1 missing length value). Hb mean concentrations between rural and peri-urban children were compared using *t*-tests and comparisons between binary variables were analyzed using Pearson chi-square tests. Hb concentrations were adjusted based on global recommendations [33]. Different superscript letters in the same row indicate statistical differences between rural and peri-urban children (*p* < 0.05).

## Data Availability

Data are available upon request from the co-authors due to ethical and privacy restrictions.
